# Peer victimization (bullying) on mental health, behavioral problems, cognition, and academic performance in preadolescent children in the ABCD Study

**DOI:** 10.3389/fpsyg.2022.925727

**Published:** 2022-09-26

**Authors:** Miriam S. Menken, Amal Isaiah, Huajun Liang, Pedro Rodriguez Rivera, Christine C. Cloak, Gloria Reeves, Nancy A. Lever, Linda Chang

**Affiliations:** ^1^Chang Laboratory, Department of Radiology and Nuclear Medicine, University of Maryland School of Medicine, Baltimore, MD, United States; ^2^Department of Otorhinolaryngology—Head and Neck Surgery, University of Maryland School of Medicine, Baltimore, MD, United States; ^3^Department of Pediatrics, University of Maryland School of Medicine, Baltimore, MD, United States; ^4^Department of Psychiatry, University of Maryland School of Medicine, Baltimore, MD, United States; ^5^Department of Neurology, University of Maryland School of Medicine, Baltimore, MD, United States; ^6^Department of Neurology, Johns Hopkins University School of Medicine, Baltimore, MD, United States

**Keywords:** peer victimization, sex differences, suicidality, bullying, internalizing and externalizing behavior

## Abstract

**Objective:**

Peer victimization is a substantial early life stressor linked to psychiatric symptoms and poor academic performance. However, the sex-specific cognitive or behavioral outcomes of bullying have not been well-described in preadolescent children.

**Methods:**

Using the baseline dataset of the Adolescent Brain Cognitive Development (ABCD) Study 2.0.1 data repository (*N* = 11,875), we evaluated associations between parent-reported bullying victimization, suicidality (suicidal ideation, intent, and/or behavior), and non-suicidal self-injury (NSSI), as well as internalizing and externalizing behavioral problems, cognition, and academic performance.

**Results:**

Of the 11,015 9-10-year-old children included in the analyses (5,263 girls), 15.3% experienced bullying victimization, as reported by the primary caregiver. Of these, boys were more likely to be bullied than girls (odds ratio [OR], 1.2 [95% CI, 1.1–1.3]; *p* = 0.004). Children who were bullied were more likely to display NSSI or passive suicidality (OR, 2.4 [95% CI, 2.0–2.9]; *p* < 0.001) and active suicidality (OR, 3.4 [95% CI, 2.7–4.2]; *p* < 0.001). Bullied children also had lower cognitive scores, greater behavioral problems, and poorer grades (*p* < 0.001). Across all participants, boys had poorer grades and greater behavioral problems than girls; however, bullied boys had greater behavioral problems than girls in several areas (*p* < 0.001). Compared to their non-bullied peers, bullied children with greater non-suicidal self-injury or suicidality also had greater behavioral problems and poorer grades (*p* < 0.001).

**Conclusion:**

These findings highlight the sex-specific effects of bullying, and the negative associations of bullying victimization with cognitive performance, behavioral problems, and academic performance. Future longitudinal studies will identify the natural history and neural correlates of these deficits during adolescence.

## Introduction

Suicide was the second leading cause of death for children aged 10–14 years in 2019 in the US (CDC, 2020b) and rates continue to rise substantially (National Center for Health Statistics, 2019). Bullying victimization was identified as a modifiable risk factor for suicide attempts in adolescence (Kim et al., 2011) or adulthood (Meltzer et al., 2011), but associations of bullying with self-harm behavior and thoughts in younger children are not well-described (Serafini et al., 2021). One study showed that both non-suicidal self-injury (NSSI; e.g., superficial cutting) and suicidality (e.g., passive and active suicidal ideation, intent, and/or behavior) were more prevalent in adults who suffered childhood bullying (Copeland et al., 2013). In addition, boys were more than twice as likely to die by suicide than girls (National Center for Health Statistics, 2019). Furthermore, the prevalence of suicidality was high among sexual minority youth (Liu et al., 2020) and in underweight or obese children (Lian et al., 2018). Therefore, understanding the interactions between these factors in a large, diverse population-based study of preadolescent children could guide the development of early detection and personalized intervention strategies.

Only one study identified an association between bullying and cognitive function, in which bullying victimization by age six was associated with lower executive function in preadolescence (Holmes et al., 2016). Being bullied was also negatively associated with academic performance (Wang et al., 2014; Mundy et al., 2017); while boys had lower grade-point averages than girls overall, this did not differ by victimization status (Wang et al., 2014). Childhood bullying victimization is also associated with internalizing symptoms (e.g., anxiety, depression), and externalizing behaviors (e.g., aggression; Copeland et al., 2013; Leiner et al., 2014; Wu et al., 2018). Only one of these studies identified a sex-specific effect, with girls showing a stronger association between bullying victimization and social anxiety than boys (Wu et al., 2018). Low IQs, deficits in cognitive or information processing abilities, attention disorders, aggression, poor behavioral control, and emotional problems are all listed by the Centers for Disease Control and Prevention (CDC) as risk factors for youth violence perpetration and victimization (CDC, 2020a). Conversely, high IQs and grade point averages are listed as protective factors against the likelihood of youth violence (CDC, 2020a). Delays in various domains of cognition, such as processing speed or inhibitory control may impact a youth’s ability to communicate and ask for help (Haigh et al., 2018) and may cause them to place themselves in risky situations (Poon, 2016). Understanding how facets of cognition, academic performance, behavioral problems, and emotional health are impacted by bullying victimization is needed to provide and implement appropriate interventions.

Overall, boys are more likely to experience bullying victimization than girls (Cook et al., 2010). However, when examining the effects of bullying in the context of sex differences in preadolescent physical, social, emotional, and cognitive development, the sex-specific effect of bullying becomes harder to define. Physically, boys are stronger (Marta et al., 2012), faster (Marta et al., 2012), and have larger brain sizes than girls (Lenroot and Giedd, 2010). Girls are more agile (Marta et al., 2012), have better balance (Marta et al., 2012), and reach peak brain volumes earlier than boys (Lenroot and Giedd, 2010). Socially, language develops earlier among girls, and boys are more likely to develop speech or language impairments (Adani and Cepanec, 2019). In the context of bullying, these physical and social differences emerge; while boys are more likely to be victims of physical aggression, girls are more likely to be victims of indirect violence, like rumors or gossip (Carrera Fernández et al., 2013).

The sex-specific effect of bullying becomes even more complex when differences in emotional development are considered. Mental health disorders (i.e., attention deficit hyperactivity disorder, conduct disorder, anxiety disorders) are more prevalent among boys during childhood and early adolescence (<13 years), but the rates are comparable between boys and girls by late adolescence, with the exception of mood and anxiety disorders (Martin and Hadwin, 2022). Adolescent girls with adverse childhood experiences are more likely to develop depression and anxiety symptoms than boys with the same level of adverse experiences (Jiang et al., 2022). As mentioned above, boys are more than twice as likely to die by suicide than girls (National Center for Health Statistics, 2019). Hence, understanding sex differences in the context of the emotional impact of bullying victimization is of critical importance.

From a cognitive or academic standpoint, girls typically perform better than boys on episodic memory (Herlitz and Yonker, 2002) and processing speed (Daseking et al., 2017) tasks, while boys outperform girls on visual processing and fluid reasoning (Daseking et al., 2017). However, these sex differences in cognitive processes do not seem to impact the effect of bullying victimization on academic performance (Wang et al., 2014). Identifying sex differences and sex-specific effects of bullying victimization among preadolescent children could facilitate better risk mitigation for childhood bullying.

Prior studies delineated the relationships between bullying, behavioral problems, and academic performance, but sex-specific effects and how they might be further impacted by NSSI and suicidality remain unclear. This study used the baseline dataset of 9 and 10-year-old children in the ABCD Study to characterize the number and socio-demographic profile of children who experienced problems with bullying, and to characterize the relationship between the above factors in a sex-specific manner. To evaluate the effect of bullying on the variables described above, participants were evaluated for cognitive performance (i.e., executive function, working memory) using the National Institutes of Health (NIH) Toolbox® Cognition Battery. Parents and caregivers took self-administered standardized questionnaires relating to suicidality and NSSI for each participant on the Kiddie Schedule for Affective Disorders and Schizophrenia (KSADS) for Diagnostic and Statistical Manual of Mental Disorders (DSM-5). Psychopathology and problem behaviors were assessed in a similar regard, with parents and caregivers completing the Child Behavioral Checklist (CBCL). We hypothesized that bullying victimization would be associated with more behavioral problems and poorer cognitive performance in preadolescents. Furthermore, we expected this association to be stronger in boys than in girls, and in children who displayed suicidality.

## Materials and methods

### Data source

We used the baseline, cross-sectional dataset (v.2.0.1) of 11,875 participants in the ABCD study, collected 9/1/2016–10/15/2018 at 21 sites across the United States. The ABCD study is an ongoing, longitudinal, observational study evaluating children starting at ages 9–10 years old and additional information from their parents/caregivers. The children were largely recruited through local elementary and charter schools, and twins were mainly recruited from birth registries (Garavan et al., 2018). The sites included in the study were specifically chosen because they encompass close to 20% of the US population of 9- and 10-year-olds and aim to represent an estimate of the national socio-demographics (Garavan et al., 2018). Participants were included if they were 9–10 years old, and able to provide written consent (parent) and assent (youth). Exclusion criteria included the youth not being proficient in English, the parent not being fluent in English or Spanish, any major neurological, medical, intellectual condition, and anything that would exclude them from getting an MRI (Thompson et al., 2019). The baseline 9–10 year-old age range was selected to fully capture any changes in physical maturation, brain morphometry, cognition, and mental health prior to and during puberty and adolescence (Thompson et al., 2019). All parent–child dyads provided written informed consent/assent. The study was approved by local (site-specific) and central (University of California, San Diego) institutional review boards. The study sample size allowed for adequate power to identify small to medium effect sizes (Garavan et al., 2018).

### Demographics

The children’s sex-assigned-at-birth, gender identity and sexual orientation, age (in months), race/ethnicity, total family income, and the participating caregiver’s educational level were collected from the caregiver self-reports.

### Bullying victimization

On the K-SADS (Kaufman et al., 2000) background items, the caregivers were asked, “Does your child have any problems with bullying at school or in your neighborhood?” A “yes” response was categorized as “*Bullied*” and a “no” response as “*Not bullied.*” Youth reports of being bullied were collected only sporadically at baseline; therefore, they were not included in this analysis.

### NSSI and suicidality

The caregiver K-SADS also assessed suicidality and NSSI. Categories were classified hierarchically based on the highest level reported. For these analyses, NSSI and passive suicidal ideation were combined into the “NSSI/Passive” category, and all active ideation, plans, or attempts were grouped into the “Active” category. For consistency in reporting, only the parent-reported K-SADS information was used. NSSI was assessed with the following questions: “*Sometimes when kids get upset or feel numb, they may do some things to hurt themselves, like scratching, cutting, or burning themselves. In the past two weeks, how often has your child done any of these things or other things to try to hurt himself or herself?*” and “*Was there ever a time in the past when your child did things to hurt himself or herself on purpose because your child was upset, like cut, scratch or burn himself or herself?*” To assess passive suicidality, the parent was asked whether their “*child wished he or she was dead or had thoughts that he or she would be better off dead?*” in the past two weeks or ever in the past. Active suicidality was assessed in a similar regard in the past two weeks or ever, with questions about the child having thoughts of wanting to kill him/herself as well as actual suicide attempts. The interrater reliability of the KSADS suicide module was moderate to strong, with a kappa of 0.9 for ideation (passive or active), 0.83 for attempts, and 0.71 for NSSI (Campos et al., 2021). For suicidal ideation, the intraclass correlation coefficient (ICC) is 0.69 for test–retest reliability and 0.70 for parent-summary agreement (Chambers et al., 1985). For number of suicide attempts, the ICCs are 0.78 for test–retest reliability and 0.74 for parent-summary agreement (Chambers et al., 1985). The overall test–retest reliability for suicidal ideation and behavior was a correlation coefficient of 0.81 (Chambers et al., 1985).

### Behavioral measures

Problem behaviors were assessed using the (CBCL; Achenbach, 2009), a validated DSM-oriented scale that assessed caregiver reported ratings of youth emotional and behavioral problems during the previous 6 months. The CBCL includes the following domains: anxious/depressed, withdrawn/depressed, somatic, social, thought, attention, rule-breaking, aggression, and grouped scores for internalizing, externalizing, and total problems. Examples of items assessed include the parent answering, “*Not True*,” “*Somewhat/Sometimes True*,” or “*Very True/Often True*” to statements about their child, like “*Acts too young for their age*,” “*Argues a lot*,” and “*There is very little that they enjoy*.” Raw scores were converted into age- and sex-based t-scores. The CBCL has a high test–retest reliability (ICC of 0.95), moderately high internal consistency (alphas ranged from 0.63 to 0.79), and cross-informant agreement between pairs of parents (mean *r*: 0.76; Achenbach and Rescorla, 2001). The stability of the scale scores for problem behaviors was significant at *p* < 0.05 at 12 months (*r*: 0.64–0.82) and 24 months (*r*: 0.50–0.82; Achenbach and Rescorla, 2001).

### Cognitive measures

General and domain-specific cognitive measures were assessed using the NIH Cognition Battery Toolbox®, a widely-used, validated assessment of cognitive performance in children (Luciana et al., 2018), which includes tests that evaluated seven domains of cognitive processes and three composite scores (Table 1). The tests administered for each domain were Picture Vocabulary (language and verbal intellect), Flanker Inhibitory Control and Attention (cognitive control and attention), List Sorting Working Memory (working memory, categorization, information processing), Dimensional Card Sort (flexible thinking, concept formation, set shifting), Pattern Comparison Processing Speed (processing speed and information processing), Picture Sequence Memory (visuospatial sequencing and memory), and Oral Reading Recognition (reading ability, language, academic achievement; Luciana et al., 2018). The three composite scores generated from these tests included Crystallized Composite (Picture Vocabulary and Oral Reading Recognition), Fluid Composite (five other tests), and Cognitive Total Composite (all seven tests).

**Table 1:** NIH toolbox cognition battery (Luciana et al., 2018).

NIH toolbox cognition battery task	Cognitive domain	
Picture vocabulary^a^	Language, Verbal Intellect	
Flanker inhibitory control and attention^b^	Cognitive Control, Attention	
List sorting working memory^b^	Working Memory, Categorization, Information Processing	
Dimensional card sort^b^	Flexible thinking, Concept Formation, Set Shifting	
Pattern comparison processing speed^b^	Processing Speed, Information Processing	
Picture sequence memory^b^	Visuospatial Sequencing, Memory	
Oral reading recognition^a^	Reading Ability, Language, Academic Achievement	

^a^Crystallized Composite	^b^Fluid Composite	Cognitive Total Composite

The NIH Toolbox Cognition Domain uses seven tests to assess five cognitive domains. The Fluid Composite score is comprised of executive function (cognitive control, attention, cognitive flexibility), working memory, processing speed, and episodic memory. The Crystallized Composite score is a measure of language (auditory comprehension and reading decoding). The Cognitive Total Composite score is made up of all seven test scores.^a^refers to Crystallized Composite.^b^refers to Fluid Composite.

The Picture Vocabulary test includes an audio of a vocabulary word, like “*ripple*,” which the participant then had to match to one of four pictures. For the Flanker Inhibitory Control and Attention test, the participant was shown a row of arrows, and had to tap a button with the arrow pointing left or right to match the direction that the middle arrow was pointing. The List Sorting Working Memory test includes a list of foods or animals being displayed and read aloud to the youth, who then must sort them into size order by memory. The Dimensional Card Sort task involves the participant switching from sorting cards one way (by color) to sorting them another way (by shape). In the Picture Sequence Memory task, the participant is shown a set of 15 pictures, and then must sort them into the order they were shown by memory. Lastly, the Oral Reading Test involves the participant reading a list of words out loud to the research assistant. The NIH Toolbox composite scores had strong test–retest correlations of *r* = 0.86 for Fluid, *r* = 0.92 for Crystallized, and r = 0.90 for Cognitive Total (all *p* < 0.001) (Heaton et al., 2014). There was also strong convergent validity for the three composite scores (Fluid: *r* = 0.78; Crystallized: *r* = 0.90; Cognitive Total: *r* = 0.89) (Heaton et al., 2014).

### Grades/academic performance

The children’s overall academic grades were reported by the caregiver. Participants marked as “ungraded” or “Not applicable” were excluded.

### Body mass index (BMI) *z*-scores

Height and weight were measured at the baseline visit (average of 2–3 measurements). BMI *z*-scores were calculated using the ‘*z-scorer’* package in R and classified into standard deviation categories classified by the World Health Organization (WHO, 2021). Height > 60.5 and < 50.3 inches, as well as weight > 129.9 and < 56.0 pounds were deemed outliers and capped at the 5 and 95% confidence interval values (*n* = 581 underweight, 594 overweight, 592 short, 641 tall).

### Statistical analysis

Descriptive statistics (means, stand deviation, frequency counts, and percentages) were used to report demographic data within each bullied-NSSI/suicidal subgroup. Chi-square tests for categorical data and t-tests for continuous variables were used to assess demographic differences across subgroups. Odds ratios were used to examine the unadjusted association between bullying and NSSI/suicidality with the covariates in this study. We used R (version 3.6.2) along with finalfit, dplyr, and ggplot2 packages to generate plots for the odds ratios and 95% confidence intervals.

We examined whether reports of being bullied, sex, NSSI, and suicidality were associated with child behavioral and emotional problems and with cognition using generalized additive models (Hastie and Tibshirani, 1986). Generalized additive models can assess the impact of non-linear effects (e.g., asymmetric categories of parent education and family income). We then investigated whether the combined effect of bullying, NSSI, and suicidality was associated with worse outcomes, i.e., higher CBCL scores and poor cognitive measures, by evaluating the significance of the interaction between these behaviors using generalized additive models while accounting for the covariates of the youth’s race, age, sex assigned at birth, caregiver education level, and family income. These demographic factors were previously associated with bullying victimization (Jansen et al., 2012; Låftman et al., 2013; Wang et al., 2020). The family/sibling ID nested by site were included as random effects. We also included BMI z-score and the sexual orientation/identity of the child in the model to determine their impact on CBCL scores. Statistical significance for all our analyses was set at *p* < 0.05 corrected for multiple comparisons (Hochberg, 1988).

## Results

### Prevalence of bullying victimization, NSSI, and suicidality in the ABCD Study

**Figure 1:**
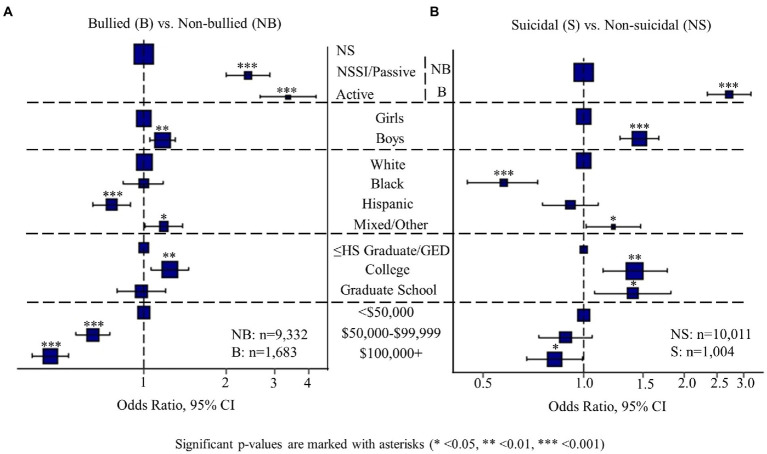
This plot displays the odds ratios for experiencing bullying problems **(A)** and suicidality **(B)**, based on various behavioral and demographic specifications.

Data from 11,015 children were included after excluding missing data (7.2%) and were grouped by reported bullying victimization status (*N* = 1,683 “bullied”; *N* = 9,332 “non-bullied”) and stratified further by 3 levels of NSSI and suicidality (Table 2). The bullied group had more boys (56.3%) and racial/ethnic minority (50.2%) children than the non-bullied group (51.5% boys, 45.6% racial/ethnic minority; *p* < 0.001). Additionally, the proportion of caregivers without college education was higher in the bullied group (17.1%) than in the non-bullied group (15.6%; *p* < 0.001). Furthermore, more families of children from the bullied group (42.3%) than those from the non-bullied group (29.3%) had an annual income of $50,000 or less (*p* < 0.001). The groups differed in all demographic categories except for age (*p* = 0.58), and income levels did not differ by NSSI and suicidality (*p* = 0.76). Bullied children were more likely to display NSSI or passive suicidality [OR, 2.4 (95% CI, 2.0–2.9); *p* < 0.001] and active suicidality [OR, 3.4 (95% CI, 2.7–4.2); *p* < 0.001] than those with no bullying (Figure 1A).

**Table 2:** An overview of sample characteristics from the ABCD Study.

	Non-bullied (*n* = 9,332, 84.7%)						Bullied (*n* = 1,683, 15.3%)						*p*-Value
	Non-suicidal *n* = 8,636 (92.5%)		NSSI/ Passive *n* = 472 (5.0%)		Active *n* = 224 (2.4%)		Non-suicidal *n* = 1,375 (81.6%)		NSSI/ Passive *n* = 183 (10.8%)		Active *n* = 125 (7.4%)		Bullied
*Sex:*	***n***	**%**	***N***	**%**	***n***	**%**	***n***	**%**	***n***	**%**	*n*	**%**	
Boys	4,384	50.8	274	58.1	147	65.6	751	54.6	112	61.2	84	67.2	<0.001
Girls	4,252	49.2	198	41.9	77	34.4	624	45.4	71	38.8	41	32.8	<0.001
*Age* (months ± standard deviations):	118.9	7.5	118.7	7.3	118.9	7.5	118.8	7.9	120.1	7.5	120.1	7.6	0.58
*Race/Ethnicity:*													
White	4,666	54.0	290	61.4	120	53.6	688	50	86	47	64	51.2	<0.001
Black	1,234	14.3	36	7.6	23	10.3	256	18.6	17	9.2	20	16	<0.001
Hispanic	1708	19.8	83	17.6	48	21.4	248	18	33	18	20	16	<0.001
Asian	185	2.1	11	2.3	5	2.2	13	0.9	4	2.1	2	1.6	<0.001
Mixed/Other	843	9.8	52	11	28	12.5	170	12.4	43	23.5	19	15.2	<0.001
*Parent education:*													
≤HS Graduate/GED	1,378	16	54	11.4	25	11.2	247	18	27	14.8	14	11.2	<0.001
Any College	4,900	56.7	283	60	140	62.5	866	63	120	65.6	85	68	<0.001
Graduate School	2,358	27.3	135	28.6	59	26.3	262	19	36	19.7	26	20.8	<0.001
*Total family income:*													
<$50,000	2,545	29.5	124	26.3	63	28.1	578	42	79	43.2	55	44	<0.001
$50,000–$99,999	2,395	27.7	133	28.2	59	26.3	375	27.3	53	29	40	32	<0.001
$100,000+	3,696	42.8	215	45.6	102	45.5	422	30.7	51	27.9	30	24	<0.001
	Non-bullied (*n* = 9,280, 84.7%)						Bullied (*n* = 1,670, 15.3%)						
*Gay/Transgender:*	***n***	**%**	***N***	**%**	***n***	**%**	***n***	**%**	***n***	**%**	***n***	**%**	
No	7,963	92.7	410	87	197	89.1	1,235	90.4	156	86.2	109	88.6	<0.001
Maybe	620	7.2	61	13	23	10.4	127	9.3	25	13.8	14	11.4	<0.001
Yes	5	0.1	0	0	1	0.5	4	0.3	0	0	0	0	<0.001
*BMI z-score:*													
Thinness: <−2.0	107	1.2	2	0.4	0	0	17	1.2	1	0.5	4	3.2	<0.001
Healthy: −2.0 ≤ ≥1.0	5,425	63.1	292	61.9	142	64.2	730	53.4	100	55.2	60	48.7	<0.001
Overweight: 1.0< >2.0	1,650	19.2	112	23.7	44	19.9	278	20.3	40	22	29	23.5	<0.001
Obese: ≥2.0	1,406	16.3	65	13.8	35	15.8	341	24.9	40	22	30	24.3	<0.001

Eight hundred and sixty participants were excluded due to missing data for any of the main variables, covariates, or CBCL data. For statistical analyses, Race/Ethnicity was categorized as White, Black, Hispanic, and other (including Mixed race and Asian).

### Sex differences in bullying, behavioral problems, and cognition

**Figure 2:**
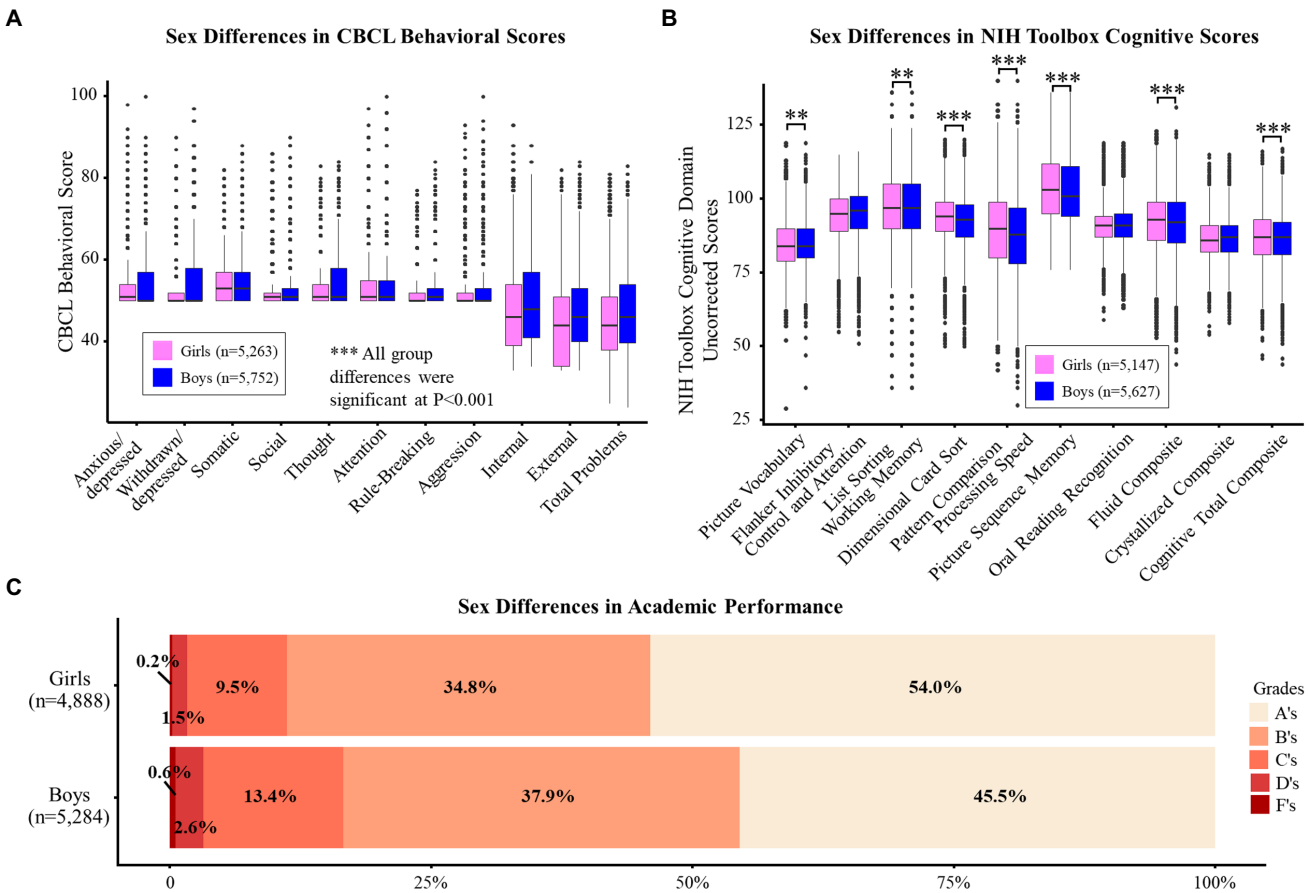
**(A,B)** display the unadjusted mean *t*-scores for each CBCL domain **(A)** and the uncorrected score for each NIH Toolbox Cognitive Domain **(B)** by sex. Significant *p*-values are marked with asterisks (* <0.05, ** <0.01, *** <0.001). **(C)** displays the distribution of caregiver-reported academic grades by bullying and sex. Chi-square tests were used to determine the differences in distributions between and within groups. All comparisons were significant.

Sex differences were observed for bullying victimization; boys were more likely to be bullied than girls, based on caregiver reports [Figure 1A; odds ratio (OR), 1.2 (95% CI, 1.1–1.3); *p* = 0.004]. NSSI and suicidality were also more prevalent amongst boys than girls [Figure 1B; OR, 1.5 (95% CI, 1.3–1.7); *p* < 0.001]. Additionally, boys scored higher than girls in all CBCL domains except somatic (Figure 2A; Supplementary Table S1; *p* < 0.001). Sex differences were also found in cognition. While boys performed better than girls on the language/verbal intellect and working memory tasks, girls performed better than boys on the executive function (flexible thinking/concept formation/set shifting), processing speed, and visuospatial sequencing/memory tasks (Figure 2B; *p* < 0.01). Girls also scored higher than boys on the Fluid (all tests except language/verbal intellect and reading ability/language/academic achievement) and Cognitive Total Composite (all seven tests) scores. Chi-square tests showed that overall, boys had lower academic grades than girls (Figure 2C; *p* < 0.001), but this effect did not differ by bullying victimization status.

### Influence of race/ethnicity and socioeconomic status on bullying, NSSI, and suicidality

Children of Hispanic race/ethnicity were less likely [OR, 0.8 (95% CI, 0.7–0.9); *p* = 0.001] to experience bullying victimization than White children. While higher caregiver education was associated with higher risk of getting bullied [OR, 1.2 (95% CI, 1.1–1.5); *p* = 0.007], higher income reduced the risk [OR, 0.5 (95% CI, 0.4–0.5); *p* < 0.001]. Like bullying, NSSI and suicidality were associated with higher parental education [OR, 1.4 (95% CI, 1.1–1.8); *p* = 0.013] and lower family income [OR, 0.8 (95% CI, 0.7–1.0); *p* = 0.042; Figure 1B].

### Behavioral outcomes of bullying

**Figure 3:**
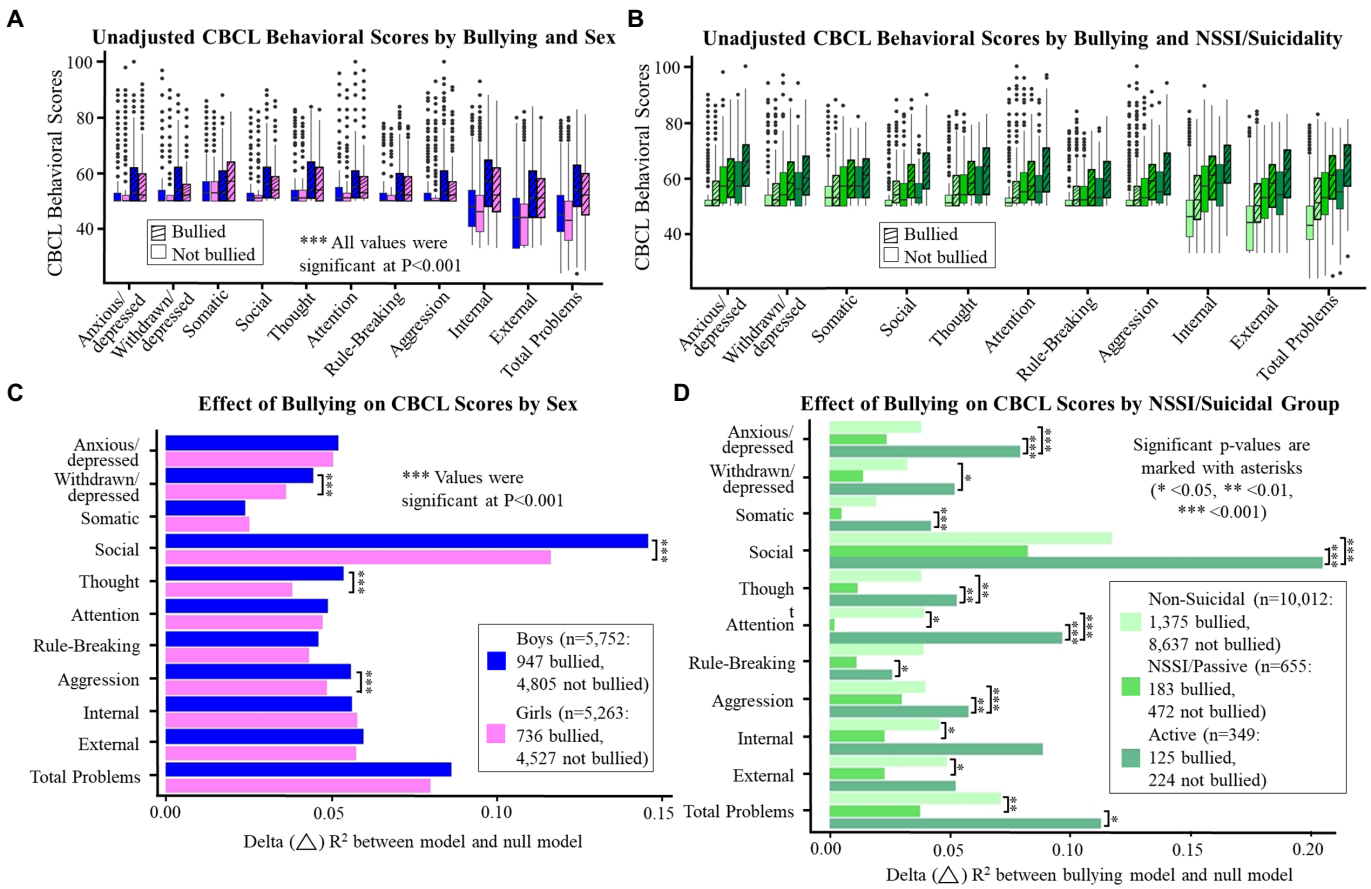
**(A–D)** display the unadjusted mean *t*-scores for each CBCL domain by reported bullying **(A,B)** and the effect sizes of the impact of bullying on CBCL domain scores **(C,D)** by sex **(A,C)** or NSSI/suicidality group **(B,D)**. Significant *p*-values are marked with asterisks (* <0.05, ** <0.01, *** <0.001).

Children who were bullied had higher CBCL *t*-scores in all domains (total problem score difference [ΔTP] boys = 9.2, *p* < 0.001; girls = 8.9, *p* < 0.001; Figure 3A: unadjusted; Figure 3C and Supplementary Table S2: adjusted). Sex was associated with variance in all CBCL domain scores in both the bullied (Δ*R*^2^ = 0.001–0.021, *p* < 0.001; not shown) and non-bullied groups (Δ*R*^2^ = 0.001–0.015, *p* < 0.001; not shown). Race and income contributed significantly to variance in CBCL *t*-scores in most of the domains, though the effect sizes were small (Δ*R*^2^ = 0.002–0.013, *p* < 0.05; Supplementary Table S2). Secondary analyses (Supplementary Table S2) showed that adding gender identity as a predictor improved model fit in nine out of ten CBCL domains (Δ*R*^2^ = 0.001–0.007, *p* < 0.05), and adding BMI improved the fit in six of ten domains (Δ*R*^2^ = 0.001–0.003, *p* < 0.05). Sex-specific effects were observed with CBCL in relation to bullying. Although the effect sizes were minimal, interactions were found between bullying status and sex in CBCL scales for withdrawn/depressed, social, thought, and aggression (Figure 3C; Δ*R*^2^ = 0.001, *p* < 0.01), with the bullied boys having greater problems than the bullied girls in each of these syndrome scales.

### Bullying, NSSI, suicidality, and behavioral problems

As shown in Figures 3B,D; Supplementary Table S1, NSSI and suicidality were associated with higher CBCL *t*-scores in both the reported bullied (ΔTP between active and non-suicidal = 14.4, *p* < 0.001) and non-bullied groups (ΔTP = 14.4, *p* < 0.001). Likelihood ratio tests show that bullying was associated with higher CBCL domain scores in the non-suicidal (Δ*R*^2^ = 0.019–0.117, *p* < 0.001; not shown) and active suicidal groups (Δ*R*^2^ = 0.028–0.214, *p* < 0.001; not shown). In the NSSI/passive suicidal group, the report of being bullied was associated with higher CBCL scores in all domains (Δ*R*^2^ = 0.005–0.081, *p* < 0.05; not shown) except attention (Δ*R*^2^ = 0.004, *p* = 0.06; not shown). We also identified a small interaction in the relationship between bullying, NSSI, and suicidality, with the bullied group having greater behavioral problems in most domains when they also endorsed active suicidal tendencies compared to the NSSI/passive youth (ΔTP = 6.1, Δ*R*^2^ = 0.001, *p* < 0.001; not shown), but this relationship was not found in the non-bullied groups (ΔTP = 2.7, Δ*R*^2^ = 0.001, *p* = 0.06; not shown). Interestingly, the impact of removing the term for NSSI/suicidal behaviors was greater in the bullied group (Δ*R*^2^ = 0.038–0.131, *p* < 0.05; not shown) than in the non-bullied group (Δ*R*^2^ = 0.024–0.081, *p* < 0.05; not shown). The NSSI/passive and active suicidal groups also differed among bullied children alone, specifically in the areas of social, thought, attention, and total problems (Bullied: *p* < 0.001; Non-bullied: *p* = 0.06–0.97; not shown).

### Cognitive outcomes of bullying

**Figure 4:**
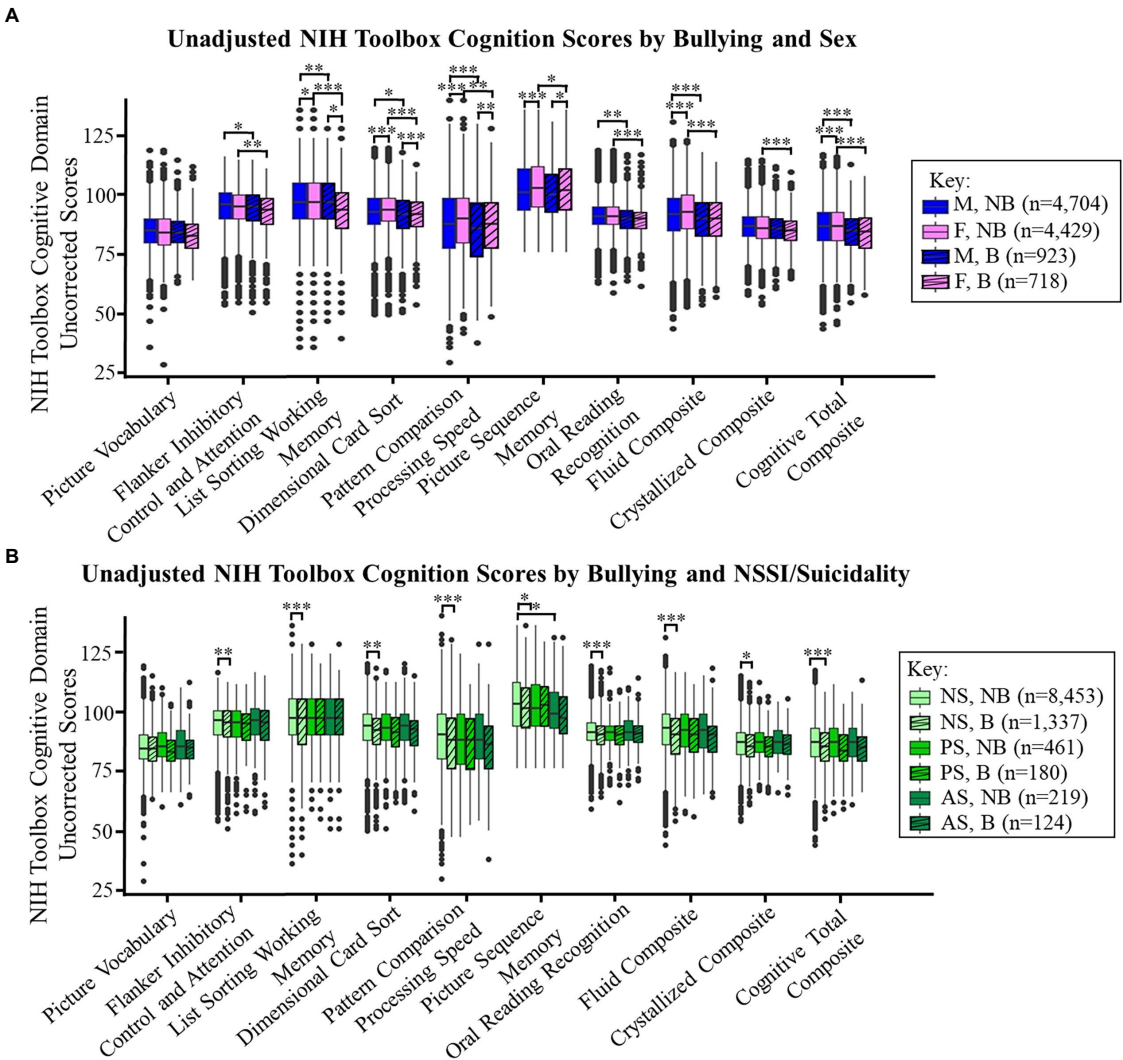
**(A,B)** display the NIH Toolbox Cognitive Domain uncorrected score means by bullying (striped) based on **(A)** sex or **(B)** by NSSI/suicidality (NS: non-suicidal, PS: NSSI or passive suicidal ideation, AS: active suicidal ideation or suicide attempts). Significant *p*-values are marked with asterisks (* <0.05, ** <0.01, *** <0.001). B, bullied and NB, non-bullied.

**Figure 5:**
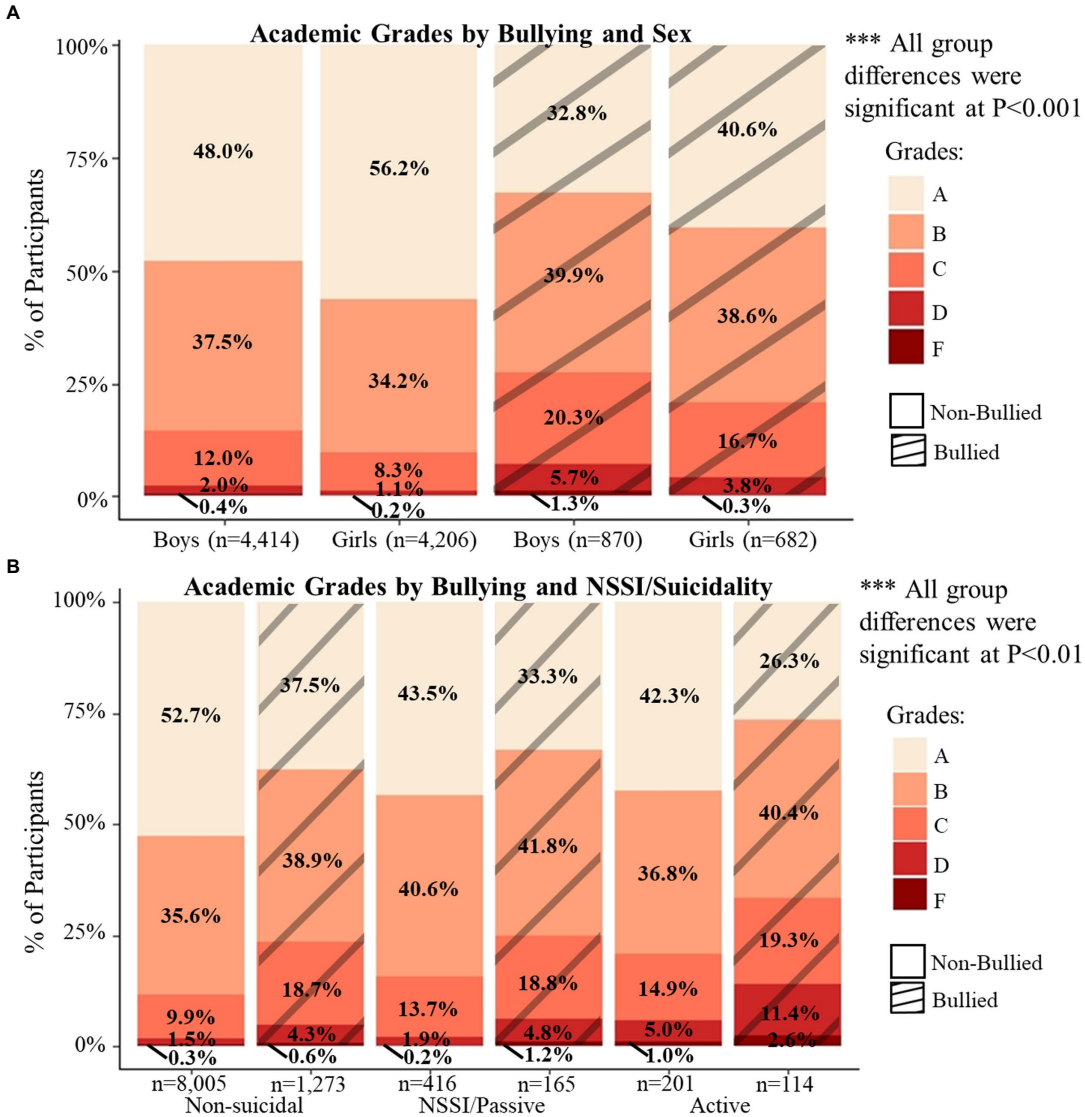
**(A,B)** display the distribution of caregiver-reported academic grades by bullying (striped) and sex **(A)** or NSSI/suicidality **(B)**. Chi-square tests were used to determine the differences in distributions between and within groups. All comparisons were significant.

Children in the bullied group had lower scores on 7 of 10 NIH Toolbox Cognitive domains than children who were in the non-bullied group (Figure 4A; cognition total difference for boys = 1.1, *p* < 0.001; girls = 1.7, *p* < 0.001; Supplementary Table S3). Sex-specific effects were observed on the cognitive measures, with girls who were bullied having lower visuospatial sequencing/memory (*p* < 0.05) and crystallized composite scores (*p* < 0.001) scores than non-bullied girls (Figure 4A). When separated into groups by NSSI, suicidality, and bullying victimization (six groups), those bullied and non-suicidal had lower scores in all domains, except the Picture Vocabulary Test, compared to those non-bullied (Figure 4B; Δ*R*^2^ = 0.001–0.004, *p* < 0.05; not shown). However, the interactions between reported bullying, NSSI, and suicidality were again not significant (*p* = 0.96). Chi-square tests showed that grades were lower amongst bullied compared to non-bullied groups (Figure 5A; p < 0.001) and children with any level of NSSI/suicidality compared to non-suicidal children (Figure 5B; *p* < 0.001; within bullied groups: *p* = 0.009, non-bullied: *p* < 0.001).

## Discussion

Our study demonstrated that preadolescent children who were reportedly bullied were more likely to display NSSI (2.4 times) or suicidality (3.4 times) than children without reported bullying. Bullied children also had lower overall cognition, specifically in the areas of working memory, processing speed, and reading. Additionally, bullied boys had greater problems than bullied girls in the areas of withdrawn/depressed, thought, social, and aggression issues, compared to non-bullied youth, who had smaller sex differences. Consistent with prior reports, bullying victimization was associated with greater behavioral problems, including internalizing, externalizing, NSSI, and suicidality, as well as lower executive function and academic performance.

### Prevalence of bullying victimization, NSSI, and suicidality in the ABCD Study

In the current study, the prevalence of caregiver-reported bullying victimization was 15.3%, which is lower than the 22.4% of children aged 6–11 reported in the National Survey of Children’s Health (Lebrun-Harris et al., 2020). Consistent with previous studies (Serafini et al., 2021), the prevalence of any NSSI or suicidality was higher among those being bullied (18.2%) compared to their non-bullied peers (7.4%). While the prevalence of these behaviors within the non-bullied group is similar to earlier studies (DeVille et al., 2020; Janiri et al., 2020), a higher prevalence in the bullied group suggests that bullying may be an important and potentially preventable risk factor for NSSI and suicidality. Additionally, since the prevalence of active suicidality peaks during mid-adolescence for girls and rises during late adolescence for boys (Boeninger et al., 2010), we can expect the rates of these suicidal intents and behaviors among bullied children to increase further as they get older.

### Sex differences in bullying, behavioral problems, and cognition

Our findings of a higher prevalence of being bullied among boys are consistent with earlier reports (Wang et al., 2020). Caregivers in our study may be more aware of bullying amongst boys, due to the greater physical manifestation, relative to girls (Silva et al., 2013). Additionally, boys are more likely to report being bullied to their parents or caregivers than girls (Silva et al., 2013). Similar to previous ABCD results (DeVille et al., 2020), we found that boys had a higher risk of NSSI and suicidality than girls. Trends in sex differences of suicidality throughout development vary over time (Boeninger et al., 2010). Therefore, future longitudinal data from the ABCD study will allow us to confirm this trajectory and to determine the potential influence of bullying on these trajectories.

Boys had greater levels of behavioral problems in all areas, except somatic, where girls scored higher than boys, which is similar to one earlier study (Kowalski et al., 2014). This finding is consistent with the more frequent reporting of pain in women than in men (Dahlhamer, 2018) and the greater prevalence of childhood mental health disorders among boys (Martin and Hadwin, 2022).

Consistent with earlier findings, boys had better language/verbal intellect than girls (Boyle, 1987), and girls performed better than boys on the processing speed (Daseking et al., 2017) and visuospatial sequencing/memory tasks (Herlitz and Yonker, 2002), and had higher academic grades overall (Voyer and Voyer, 2014). We identified that boys performed better than girls on the working memory task, which is inconsistent with the previous findings that women outperform men on visual working memory tests (Harness et al., 2008) and no sex differences were found in verbal working memory (Harness et al., 2008; Tulsky et al., 2014). However, our result was consistent with that in a prior ABCD paper (Assari et al., 2021). These disparate findings demonstrate that sex differences in working memory performance differ in preadolescent children compared to adults (Harness et al., 2008; Tulsky et al., 2014). We also found that girls performed better than boys on the executive function task, which contradicts a recent review paper that found little support for overall sex or gender differences in executive function throughout the lifespan (Grissom and Reyes, 2019). However, regarding specific components of executive function, such as attention and impulsivity, girls aged 8 and 10 perform better than boys (Barnett et al., 2007), although this sex difference in attention may disappear in adolescence (Lange et al., 2014). Lastly, we found that girls had greater fluid and cognitive total composite scores than boys. Our results are consistent with the theory of sex differences in the maturation of intelligence; sex differences in intelligence are negligible until the age of 8, girls have an advantage from 9 until about 12, intelligence is similar until 15, and boys score higher than girls after 15 (Lynn and Irwing, 2004). Future follow-up within the ABCD Study will need to further validate this theory of the sex differences in the maturation of intelligence.

### Influence of race/ethnicity and socioeconomic status on bullying, NSSI, and suicidality

Our findings of higher prevalence of being bullied among those with a lower family income (Jansen et al., 2012), who are overweight/obese (Lian et al., 2018), and are sexual minority (Liu et al., 2020), and a lower prevalence among Hispanic children (Wang et al., 2020) are consistent with earlier reports. The lower risk for bullying among Hispanic children is likely due to their larger family networks, greater levels of social support (Landale et al., 2006), and strong cultural, family, and ethnic values within the community (Xu et al., 2020). Our finding of a greater likelihood of bullying victimization among youth from a Mixed/Other race may be impacted by other adverse circumstances (i.e., home, school, community environments) associated with a minority background. Additionally, youth from a Mixed-race are at a higher risk for poorer health, smoking, and drinking (Udry et al., 2003), possibly due to the stress related to identity conflict and not having the same sense of community compared to those from one race only (Udry et al., 2003).

The greater likelihood of children with a college-educated parent being bullied contrasts with prior studies that either found no association (Rodríguez-Enríquez et al., 2019) or an inverse association between parental education and the risk of cyberbullying victimization (Låftman et al., 2013). It is possible that the difference between bullying reports by parents with a graduate level and college education may be due to differences in the perception of being bullied, as opposed to attributing the behavior to typical preadolescent physical or verbal interactions.

We found that Black children were at a lower risk for NSSI and suicidality than White children, regardless of bullying status, which is mostly consistent with findings in older adolescents (Ivey-Stephenson, 2020). However, Black adolescents showed the highest prevalence of suicide attempts compared to White and Hispanic adolescents (Ivey-Stephenson, 2020), which we did not find among preadolescents. Consistent with previous ABCD results (Janiri et al., 2020), we found that children of caregivers with higher education also had a greater prevalence of suicidal ideation, which may be explained by a greater parental awareness with rising educational status. We also confirmed that high family income ($100,000+) was associated with lower prevalence of any level of NSSI or suicidal behaviors when compared to low family income (<$50,000; DeVille et al., 2020).

### Sex-specific behavioral outcomes of bullying

Bullied children had greater internalizing and externalizing behavioral problems than non-bullied children, which is consistent with earlier studies (Kowalski et al., 2014; Leiner et al., 2014; Wu et al., 2018). In contrast to prior reports that adolescent girls are more likely to develop depression and anxiety following adverse childhood events (Jiang et al., 2022) or bullying (Sapouna and Wolke, 2013), in our bullied group, boys had greater behavioral problems than girls in the domains of withdrawn/depressed, social, thought, and aggression. Several reasons exist for these opposite results. First, in girls, rates of depression rise significantly at the beginning of puberty (McGuire et al., 2019). Therefore, the full effect of bullying may not yet be apparent among preadolescent girls. Second, because peers react more negatively to boys who experience social problems (i.e., shy and anxiously withdrawn) than girls (Rubin and Barstead, 2014), boys with problems in these areas may have been specifically targeted by bullies. Additionally, boys who display aggressive and less social behavior were more likely to be bullied (Sugimura et al., 2017).

These studies demonstrate the importance of considerations for the sex differences in the reaction of a preadolescent to being bullied, as well as peer reactions that may differ based on the bullied youth’s sex. Intervention strategies should also consider our finding of greater levels of behavioral problems among minority races/ethnicities and lower-income families. Since minority and lower-income groups have poorer access to mental health care (McGuire and Miranda, 2008), the impact of bullying victimization on the mental health in these communities may be more debilitating than those from more advantaged backgrounds.

### Bullying, NSSI, suicidality, and behavioral problems

No prior study evaluated how bullying, NSSI, and suicidality might interact to produce behavioral problems. Our study suggests that reported bullying strongly predicted problem behaviors, especially in those with active suicidal ideation or suicide attempts. However, among those with NSSI behaviors or passive suicidal ideation, the effect of reported bullying victimization on problem behaviors was smaller compared with those deemed non-suicidal. Thus, children who were bullied, but display NSSI or passive suicidality, might be less likely to present easily recognized problematic behaviors; therefore, their mental health disruptions might be underestimated by caregivers or teachers.

### Cognitive outcomes of bullying

Children in the reported bullying group had lower executive function (Holmes et al., 2016) and fluid composite (Huepe et al., 2011) scores than children in the non-bullied group. While the effect sizes were small, we identified differences in the areas of working memory, processing speed, reading, and in the cognition total composite score. An important risk factor for peer victimization is executive function, which was linked to social competence (Alduncin et al., 2014), the ability to read and understand social cues. As children mature, the resulting low peer acceptance may place them at risk to be either a victim or bully, while their poor executive functioning may also be detrimental to their academic performance. Furthermore, internalizing or externalizing problems may lead to both academic and social struggles in formal educational settings (Hay et al., 2004). Our novel findings of altered cognitive scores in several areas aside from executive function have important implications and will need to be explored further.

Similar to previous studies (Wang et al., 2014; Mundy et al., 2017), we found that bullied children had poorer academic performance than non-bullied children. We also confirmed the finding that academic performance was lower in children who display NSSI (Rahman et al., 2018), and importantly, even lower in children who were bullied and displayed these behaviors. While academic performance is not a complete representation of overall or domain-specific intelligence, specific cognitive tests, such as reading, attention, or vocabulary, could contribute to improved or worsened academic performance (Weintraub et al., 2013). However, the associations between bullying and academic or cognitive performance are complex and involve many potential covariates or modulators, such as self-esteem, motivation, and academic engagement (Samara et al., 2021).

### Sex-specific cognitive outcomes of bullying

We identified a novel sex-specific effect, where girls who reported that they were bullied had lower episodic memory and crystallized composite scores than non-bulled girls. Episodic memory contains our memories of every day events, and is localized to the hippocampus (Dickerson and Eichenbaum, 2010), a region that displayed structural alterations in adults who were bullied (Nolfe et al., 2018). Since girls have higher perceived levels of stress than boys (Graves et al., 2021), which can negatively impact learning and memory (Vogel and Schwabe, 2016), girls in the bullied group may perceive greater stress from the bullying victimization, which in turn may affect their episodic memory. Our finding of lower crystallized composite scores (comprised of reading and vocabulary scores) among girls in the bullied group compared to the non-bullied group was similar to an earlier study by Mundy et al. (Mundy et al., 2017), who found that 8-9-year-old girls who were bullied had 6–9 month delays in reading, writing, and grammar. Our novel findings of the sex-specific effects of bullying on episodic memory, reading, and vocabulary, more in girls than in boys, should also be noted when designing intervention strategies for preadolescent children in a school setting.

### Study limitations

One limitation of the current study is that information on bullying victimization from the baseline ABCD dataset is captured solely by the presence or absence of caregiver-reported victimization because the children were at the young ages of 9–10 years. Behavioral associations found in this study might be inflated, because they were taken from concerning parents. Another limitation is the cross-sectional design, which could not provide directionality in the relationship between bullying victimization and cognitive or behavioral outcomes. Future follow-up studies will allow the evaluation of the frequency and intensity of bullying victimization and perpetration when the children are old enough to provide accurate self-reports (ages ≥11 years).

## Conclusion

Our study identified several novel findings on the sex-specific relationships between bullying, NSSI and suicidality, behavioral problems, and cognition. We also validated findings from several earlier studies, using this large dataset from a national sample to provide rigor and reproducibility. Further longitudinal studies are needed to determine whether childhood bullying is associated with increased behavioral problems and disruption in cognitive development into adolescence, and how the frequency and intensity of bullying can impact these outcomes. Approaches to prevent bullying and mitigate its negative impact must keep in mind the sex of the youth. Preadolescent bullying may be affecting problem behaviors more among boys and cognitive or academic performance more among girls. It is also important to consider that youth from racial minority or low-income backgrounds may have poorer access to mental health or academic services. Our findings highlight the negative sex-specific impact of bullying and encourage future studies to search for factors that might promote resilience to or prevent bullying.

## Data availability statement

Publicly available datasets were analyzed in this study. This data can be found at: http://dx.doi.org/10.15154/1520784.

## Ethics statement

The studies involving human participants were reviewed and approved by Institutional Review Boards (UCSD for all ABCD sites and site-specific). Written informed consent to participate in this study was provided by the participants’ legal guardian/next of kin.

## Author contributions

MM: responsible for the integrity of the data and the accuracy of the data analysis. MM, LC, AI, and CC: conceptualization. MM, PR, HL, and CC: drafting of the manuscript. MM and PR: statistical analysis. LC: obtained funding. All authors had access to the dataset used in the current study, acquisition, analysis, or interpretation of data, and critical revision of the manuscript for important intellectual content. All authors contributed to the article and approved the submitted version.

## Funding

National Institutes of Health and others. Data used in the preparation of this article were obtained from the Adolescent Brain Cognitive Development^SM^ (ABCD) Study (https://abcdstudy.org), held in the NIMH Data Archive (NDA). This is a multisite, longitudinal study designed to recruit more than 10,000 children aged 9–10 and follow them over 10 years into early adulthood. The ABCD Study® is supported by the National Institutes of Health and additional federal partners under award numbers U01DA041048, U01DA050989, U01DA051016, U01DA041022, U01DA051018, U01DA051037, U01DA050987, U01DA041174, U01DA041106, U01DA041117, U01DA041028, U01DA041134, U01DA050988, U01DA051039, U01DA041156, U01DA041025, U01DA041120, U01DA051038, U01DA041148, U01DA041093, U01DA041089, U24DA041123, U24DA041147. A full list of supporters is available at https://abcdstudy.org/federal-partners.html. A listing of participating sites and a complete listing of the study investigators can be found at https://abcdstudy.org/consortium_members/. ABCD consortium investigators designed and implemented the study and/or provided data but did not necessarily participate in the analysis or writing of this report. This manuscript reflects the views of the authors and may not reflect the opinions or views of the NIH or ABCD consortium investigators. The ABCD data used in this report came from Annual Curated Release 2.0 [https://doi.org/10.15154/1520784].

## Conflict of interest

The authors declare that the research was conducted in the absence of any commercial or financial relationships that could be construed as a potential conflict of interest.

## Publisher’s note

All claims expressed in this article are solely those of the authors and do not necessarily represent those of their affiliated organizations, or those of the publisher, the editors and the reviewers. Any product that may be evaluated in this article, or claim that may be made by its manufacturer, is not guaranteed or endorsed by the publisher.

## Abbreviations

ABCD Study: Adolescent brain cognitive development study
NSSI: Non-suicidal self-injury
OR: Odds ratio
K-SADS: Kiddie schedule for affective disorders and schizophrenia
CBCL: Child behavior checklist
NIH: National institutes of health
BMI: Body mass index
TP: Total problem score.
