# Supplementary material for: Peer victimization (bullying) on mental health, behavioral problems, cognition, and academic performance in preadolescent children in the ABCD Study
Source: Front Psychol. 2022 Sep 26;13:925727. doi: 10.3389/fpsyg.2022.925727 (PMC9549775; doi:10.3389/fpsyg.2022.925727)
Supplement: Supplementary file 1 [file Table_1.docx]

**Table S1.** CBCL marginal mean t-scores by reported bullying, sex, and suicide groups

| CBCL Area | Sex | Male  Mean [95% CI] | Female  Mean [95% CI] | Non-suicidal  Mean [95% CI] | NSSI/Passive  Mean [95% CI] | Active  Mean [95% CI] |
| --- | --- | --- | --- | --- | --- | --- |
| Anxious/depressed | Non-bullied: | 53.5 [53.0, 54.1] | 52.8 [52.3, 53.3] | 53.0 [52.6, 53.3] | 57.6 [55.2, 60.0] | 60.7 [56.7, 64.7] |
|  | Bullied: | 57.4 [56.8, 58.0] | 56.5 [55.9, 57.2] | 55.9 [55.5, 56.3] | 60.7 [58.2, 63.2] | 67.0 [62.9, 71.1] |
| Withdrawn/depressed | Non-bullied: | 55.0 [54.5, 55.6] | 53.4 [52.9, 53.8] | 54.4 [54.0, 54.7] | 60.2 [58.0, 62.4] | 62.2 [58.9, 65.5] |
|  | Bullied: | 58.7 [58.0, 59.3] | 56.1 [55.6, 56.7] | 57.1 [56.7, 57.5] | 62.5 [60.2, 64.8] | 66.4 [63.2, 69.8] |
| Somatic | Non-bullied: | 54.6 [54.1, 55.1] | 55.6 [55.0, 56.1] | 54.5 [54.1, 54.9] | 57.0 [54.9, 59.1] | 60.4 [57.3, 63.4] |
|  | Bullied: | 57.1 [56.5, 57.7] | 58.4 [57.7, 59.0] | 56.8 [56.4, 57.3] | 58.4 [56.2, 60.6] | 63.9 [60.8, 67.0] |
| Social | Non-bullied: | 53.2 [52.9, 53.6] | 52.5 [52.2, 52.9] | 52.9 [52.6, 53.1] | 57.0 [55.2, 58.7] | 57.5 [54.6, 60.3] |
|  | Bullied: | 58.4 [58.0, 58.9] | 56.8 [56.4, 57.3] | 57.0 [56.7, 57.4] | 61.4 [59.6, 63.3] | 65.3 [62.4, 68.2] |
| Thought | Non-bullied: | 54.6 [54.1, 55.1] | 53.6 [53.2, 54.1] | 54.1 [53.7, 54.4] | 60.1 [57.9, 62.3] | 61.4 [58.0, 64.7] |
|  | Bullied: | 58.5 [57.9, 59.2] | 56.7 [56.1, 57.3] | 57.1 [56.7, 57.5] | 62.2 [59.9, 64.5] | 65.8 [62.4, 69.3] |
| Attention | Non-bullied: | 54.3 [53.7, 54.8] | 53.9 [53.4, 54.4] | 53.9 [53.5, 54.3] | 60.2 [57.9, 62.6] | 59.6 [55.7, 63.6] |
|  | Bullied: | 58.2 [57.6, 58.8] | 57.5 [56.9, 58.1] | 57.1 [56.7, 57.5] | 61.6 [59.2, 64.1] | 66.6 [62.6, 70.6] |
| Rule-Breaking | Non-bullied: | 53.7 [53.3, 54.1] | 53.2 [52.8, 53.6] | 53.1 [52.8, 53.4] | 58.4 [56.7, 60.2] | 62.0 [59.0, 64.9] |
|  | Bullied: | 56.7 [56.2, 57.2] | 55.9 [55.5, 56.4] | 55.6 [55.3, 56.0] | 60.1 [58.3, 62.0] | 64.8 [61.8, 67.8] |
| Aggression | Non-bullied: | 54.1 [53.6, 54.6] | 52.8 [52.3, 53.2] | 53.3 [53.0, 53.6] | 58.5 [56.3, 60.7] | 63.9 [60.2, 67.6] |
|  | Bullied: | 58.0 [57.4, 58.6] | 55.9 [55.4, 56.4] | 56.1 [55.7, 56.4] | 61.7 [59.5, 64.0] | 69.0 [65.2, 72.8] |
| Internal | Non-bullied: | 49.3 [48.4, 50.1] | 48.0 [47.0, 48.9] | 48.5 [47.9, 49.2] | 58.4 [55.5, 61.4] | 61.7 [57.5, 65.9] |
|  | Bullied: | 56.1 [55.1, 57.2] | 55.2 [54.1, 56.3] | 54.7 [53.9, 55.5] | 62.3 [59.2, 65.4] | 68.8 [64.5, 73.1] |
| External | Non-bullied: | 47.5 [46.7, 48.4] | 46.3 [45.4, 47.1] | 46.6 [46.0, 47.2] | 56.9 [54.0, 59.8] | 62.0 [57.7, 66.4] |
|  | Bullied: | 54.7 [53.6, 55.7] | 53.1 [52.1, 54.1] | 52.9 [52.1, 53.6] | 60.7 [57.7, 63.8] | 67.8 [63.3, 72.3] |
| Total Problems | Non-bullied: | 47.1 [46.2, 48.1] | 45.6 [44.6, 46.5] | 46.3 [45.6, 47.0] | 58.0 [55.2, 60.8] | 60.7 [56.4, 64.9] |
|  | Bullied: | 56.3 [55.2, 57.5] | 54.5 [53.4, 55.6] | 54.5 [53.7, 55.4] | 62.8 [59.9, 65.8] | 68.9 [64.6, 73.3] |

Table S1 Legend: This table shows the marginal mean t-score and 95% confidence interval (CI) for each CBCL domain by sex and NSSI or suicidal category. Marginal means includes adjustment for age, race, participating parent education level, total family income level, family ID and site. The suicidal group scores also include adjustment for sex.
