# Supplementary material for: Peer victimization (bullying) on mental health, behavioral problems, cognition, and academic performance in preadolescent children in the ABCD Study
Source: Front Psychol. 2022 Sep 26;13:925727. doi: 10.3389/fpsyg.2022.925727 (PMC9549775; doi:10.3389/fpsyg.2022.925727)
Supplement: Supplementary file 2 [file Table_2.docx]

**Table S2.** Effect sizes for impact of covariates on CBCL scores by sex (ΔR^2^ values)

| CBCL Area | Sex | Age | Race | Parent  Education | Income | Site | Gay/  Trans | BMI  z-score |
| --- | --- | --- | --- | --- | --- | --- | --- | --- |
| Anxious/  depressed | Male: | 0 | **0.007** | **0.003** | **0.004** | 0 | **0.004** | 0 |
|  | Female: | 0 | **0.007** | 0.001 | **0.003** | 0 | *0.001* | 0 |
| Withdrawn/  depressed | Male: | 0 | *0.002* | 0.002 | **0.012** | 0 | **0.005** | 0 |
|  | Female: | 0 | *0.002* | 0 | **0.007** | 0 | **0.004** | *0.001* |
| Somatic | Male: | 0 | **0.003** | **0.002** | **0.006** | 0 | **0.004** | **0.003** |
|  | Female: | 0.001 | **0.005** | 0.001 | **0.005** | 0 | 0.001 | *0.001* |
| Social | Male: | 0 | 0 | 0.002 | **0.009** | 0 | **0.004** | 0.001 |
|  | Female: | 0 | 0 | *0.001* | **0.006** | 0 | 0.001 | 0.001 |
| Thought | Male: | 0 | **0.004** | **0.003** | **0.006** | 0 | **0.005** | 0 |
|  | Female: | 0 | **0.006** | *0.001* | 0.002 | 0 | **0.005** | 0 |
| Attention | Male: | 0 | 0 | 0.002 | **0.008** | 0 | **0.002** | 0.002 |
|  | Female: | 0 | 0.001 | 0.001 | **0.003** | 0 | **0.003** | 0.001 |
| Rule-Breaking | Male: | 0 | **0.006** | **0.003** | **0.013** | 0 | 0.001 | 0 |
|  | Female: | 0 | **0.003** | 0.002 | **0.008** | 0 | **0.004** | 0 |
| Aggression | Male: | *0.001* | 0.002 | 0.001 | **0.012** | 0 | 0.001 | 0 |
|  | Female: | 0 | 0.001 | 0.001 | **0.005** | 0 | 0.001 | *0.001* |
| Internal | Male: | 0 | **0.008** | **0.003** | **0.007** | 0 | **0.007** | 0 |
|  | Female: | 0 | **0.010** | 0 | **0.007** | 0 | **0.004** | 0 |
| External | Male: | 0.001 | 0 | 0.002 | **0.012** | 0 | **0.002** | 0 |
|  | Female: | 0 | *0.001* | *0.001* | **0.008** | 0 | **0.004** | 0 |
| Total Problems | Male: | *0.001* | 0.002 | **0.003** | **0.010** | <0.005 | **0.006** | 0 |
|  | Female: | 0 | **0.004** | 0.001 | **0.009** | 0 | **0.007** | 0.001 |

Table S2 Legend: This table displays the effect sizes (ΔR^2^ values) of the removal of each covariate on CBCL t-scores. Effect sizes were calculated using the difference between the main GAM model and that same model without each covariate. *All bolded R^2^ values are significant at P-value of <0.001, those underlined are significant at <0.01, and those italicized are significant at <0.05; calculated using likelihood ratio tests between main and null models and adjusted for multiple comparisons.
