# Supplementary material for: Peer victimization (bullying) on mental health, behavioral problems, cognition, and academic performance in preadolescent children in the ABCD Study
Source: Front Psychol. 2022 Sep 26;13:925727. doi: 10.3389/fpsyg.2022.925727 (PMC9549775; doi:10.3389/fpsyg.2022.925727)
Supplement: Supplementary file 3 [file Table_3.docx]

**Table S3.** NIH Toolbox Cognitive Domain Scores by Bullying and Sex

| Cognitive Domain | Sex | Non-bullied  Mean [95% CI] | Bullied  Mean [95% CI] | P-value | | | |
| --- | --- | --- | --- | --- | --- | --- | --- |
|  |  |  |  | Bullying | Sex | | Interaction |
| Picture Vocabulary Test | Male: | 82.0 [81.4, 82.6] | 82.0 [81.3, 82.7] | 0.79 | NB: | 0.07 | 0.48 |
|  | Female: | 81.0 [80.4, 81.6] | 80.7 [79.9, 81.5] | 0.44 | B: | 0.07 |  |
| Flanker Inhibitory Control and Attention Test | Male: | 91.9 [91.2, 92.7] | 91.2 [90.3, 92.2] | **0.038** | NB: | 0.37 | 0.48 |
|  | Female: | 91.8 [91.0, 92.6] | 90.8 [89.9, 91.7] | **0.006** | B: | 0.27 |  |
| List Sorting Working Memory Test | Male: | 93.7 [92.7, 94.7] | 92.5 [91.3, 93.7] | **0.009** | NB: | **0.031** | 0.40 |
|  | Female: | 91.9 [90.9, 92.9] | 90.0 [88.8, 91.3] | **<0.001** | B: | **0.040** |  |
| Dimensional Change Card Sort Test | Male: | 89.9 [89.1, 90.8] | 89.1 [88.1, 90.0] | **0.020** | NB: | **<0.001** | 0.40 |
|  | Female: | 91.4 [90.6, 92.2] | 89.7 [88.8, 90.7] | **<0.001** | B: | **<0.001** |  |
| Pattern Comparison Processing Speed Test | Male: | 85.5 [84.3, 86.8] | 83.4 [81.9, 84.9] | **<0.001** | NB: | **<0.001** | 0.64 |
|  | Female: | 88.3 [87.1, 89.6] | 86.6 [85.1, 88.1] | **0.006** | B: | **0.004** |  |
| Picture Sequence Memory Test | Male: | 100.5 [99.5, 101.5] | 99.7 [98.5, 100.9] | 0.08 | NB: | **<0.001** | 0.56 |
|  | Female: | 101.5 [100.5, 102.6] | 100.4 [ 99.2, 101.7] | **0.018** | B: | **0.048** |  |
| Oral Reading Recognition Test | Male: | 87.8 [87.3, 88.4] | 87.1 [86.5, 87.8] | **0.005** | NB: | 0.70 | 0.40 |
|  | Female: | 87.5 [87.0, 88.1] | 86.2 [85.6, 86.9] | **<0.001** | B: | 0.97 |  |
| Cognition Fluid Composite Score | Male: | 88.1 [87.2, 88.9] | 86.5 [85.5, 87.5] | **<0.001** | NB: | **<0.001** | 0.48 |
|  | Female: | 89.0 [88.1, 89.8] | 86.9 [85.9, 88.0] | **<0.001** | B: | 0.30 |  |
| Crystallized Composite Score | Male: | 83.4 [82.9, 83.9] | 83.0 [82.4, 83.6] | 0.11 | NB: | 0.40 | 0.40 |
|  | Female: | 82.7 [82.2, 83.3] | 81.9 [81.3, 82.5] | **0.001** | B: | 0.07 |  |
| Cognition Total Composite Score | Male: | 82.3 [81.6, 83.0] | 81.2 [80.4, 82.0] | **<0.001** | NB: | **<0.001** | 0.40 |
|  | Female: | 82.5 [81.8, 83.2] | 80.8 [80.0, 81.6] | **<0.001** | B: | 0.97 |  |

Table S3 Legend: This table displays the NIH Toolbox Cognitive Domain marginal means of the uncorrected standard scores for male and female participants in the bullied (B) and non-bullied (NB) groups. Means were adjusted for age, race/ethnicity, parent education, total income, family ID, and site. A generalized additive model (GAM) was used to determine the impact of bullying, sex, and their interaction (*) on each cognitive domain. These values were calculated using an outcome of each log-transformed cognitive domain, factoring in the main term, as well as the covariates listed above. Likelihood ratio tests adjusted for multiple comparisons were used to calculate the p-values above (significant values are in **bold**). The effect sizes for each domain were minimal (delta R^2^ <0.01, not shown).
